# Human Plasma Significantly Reduces Bacteriophage Infectivity Against Staphylococcus aureus Clinical Isolates

**DOI:** 10.7759/cureus.23777

**Published:** 2022-04-03

**Authors:** Prajakta Shinde, Nicholas Stamatos, James B Doub

**Affiliations:** 1 Infectious Diseases, University of Maryland School of Medicine, Baltimore, USA

**Keywords:** plasma proteins, bacteremia, periprosthetic joint infection, staphylococcus aureus, bacteriophage therapy

## Abstract

Bacteriophage therapy has been regaining interest as a potential therapeutic in treating a wide range of infections. However, there is a paucity of knowledge regarding numerous aspects of bacteriophage therapy, thereby hindering the development of proper treatment protocols and effective clinical trials. In this report, the activities of three bacteriophages are evaluated against clinical bacterial isolates in the presence and absence of human plasma (HP). The bacteriophages used in this experiment were residual therapeutic doses from the United States Food and Drug Administration (FDA) approved compassionate use cases to treat recalcitrant prosthetic joint infections (PJIs). Herein we demonstrate that in the presence of HP, the infectivity of these Staphylococcal bacteriophages was significantly reduced compared to the infectivity in the absence of HP. Inhibition of infectivity ranged from 48% to 81% for two methicillin-resistant* Staphylococcus aureus* (MRSA) clinical isolates independently infected with the same bacteriophage and 98% for a third MRSA clinical isolate infected with a different bacteriophage. In contrast, bacteriophage infectivity of an *Enterococcus faecalis* clinical isolate was not affected by the presence of HP. We hypothesize that the inhibition is correlated with plasma proteins binding to Staphylococcal surface proteins masking the receptors associated with bacteriophage attachment, thereby reducing infectivity. This has clinical ramifications for bacteriophage therapy use in treating Staphylococcal bacteremia and periprosthetic joint infections.

## Introduction

Bacteriophage therapy is a promising therapeutic adjuvant in treating multidrug-resistant bacterial infections and chronic biofilm infections. While bacteriophage therapy has been used in eastern Europe for close to 100 years, many aspects of this therapeutic are still unknown limiting the ability to devise effective protocols [1]. Consequently, the clinical trials that have been conducted have yet to prove the effectiveness of bacteriophage therapy over placebos [2]. Despite these limitations, momentum is growing for the use of bacteriophage therapy in various infections including bacteremia, prosthetic joint infections (PJIs), and cystic fibrosis pulmonary infections [3-5]. However, the narrow spectrum of activity and regulatory hurdles render only some bacterial species as potential targets currently. 

*Staphylococcus aureus* is the causative agent in numerous infectious syndromes associated with significant morbidity, mortality and immense financial ramifications, making it an attractive pathogen for pharmaceutical companies [6]. S. aureus infections are challenging to treat given numerous virulence factors and the ability of this pathogen to form hardy biofilms that conventional antibiotics have limited abilities to eradicate [7]. One specific virulence factor that has been recently proposed to cause chronic Staphylococcal infections is the formation of plasma protein aggregates that are impervious to conventional dosing of systemic antibiotics [8-10]. These add to the difficulty in treating S. aureus bacteremia and PJIs [8-9]. Unfortunately, while in vitro bacteriophage studies have shown effectiveness in reducing planktonic and biofilm infections, extrapolating these findings to in vivo use is cumbersome given the lack of knowledge on the activity of bacteriophage therapy in the presence of human plasma (HP) [11]. Therefore, our objective was to evaluate the activity of several different bacteriophages against known sensitive clinical isolates in the presence of HP.

## Materials and methods

Materials

All three bacteriophages used in this experiment were previously used to treat recalcitrant PJIs through the FDA-approved expanded access pathway and were the residual bacteriophages preserved from these cases (FDA IND’s 27458, 27513, 19274). These bacteriophages were internally named at the University of Maryland: Enterococcus phage 1 (titer: 1×1010 PFU per mL), Staphylococcal phage 1 (titer: 1×1010 PFU per mL), and Staphylococcal phage 3 (titer 2.7×109 PFU per mL). All phages were amplified and purified for clinical use as documented elsewhere [12]. Titers of bacteriophages were not altered from those used in the expanded access cases. The clinical bacterial isolates used in this experiment were the same bacteria utilized to create the bacteriophage therapies for the FDA-approved expanded access PJI cases. These included three different methicillin-resistant Staphylococcus aureus (MRSA) and one *Enterococcus faecalis* isolates. All bacteria were initially grown in brain heart infusion (BHI) broth (Becton Dickinson, Sparks, MD) and where indicated, grown with heparinized single donor HP (Innovative Research, Novi, MI). Experiments were conducted in Falcon 96 well microtiter plates.

Bacteriophage activity with and without human plasma

Overnight cultures of all four bacteria grown in BHI broth were diluted to 1% in BHI broth and were then grown to an optical density (OD) 0.30-0.60, representing exponential growth (optical density, OD 620 nm; Emax plus molecular devices plate reader). Bacterial cell cultures were then diluted to OD of 0.024 in BHI and 50 µL was added to wells of microtiter plates that contained 100 µL of BHI with or without 10% HP. Wells were infected with 50 µL of bacteriophage where indicated. Negative controls included BHI media with and without HP with normal saline and/or bacteriophages. OD was read at time zero and again after microwell plates were incubated at 37ºC for 24 h. The experiment was conducted three times and the results are the average of eight replicate samples conducted for each condition. Comparisons between the means were analyzed using two-tailed t-tests using https://www.medcalc.org. Statistical significance was determined with a p-value less than 0.05 at a 95% confidence interval.

## Results

**Figure 1:**
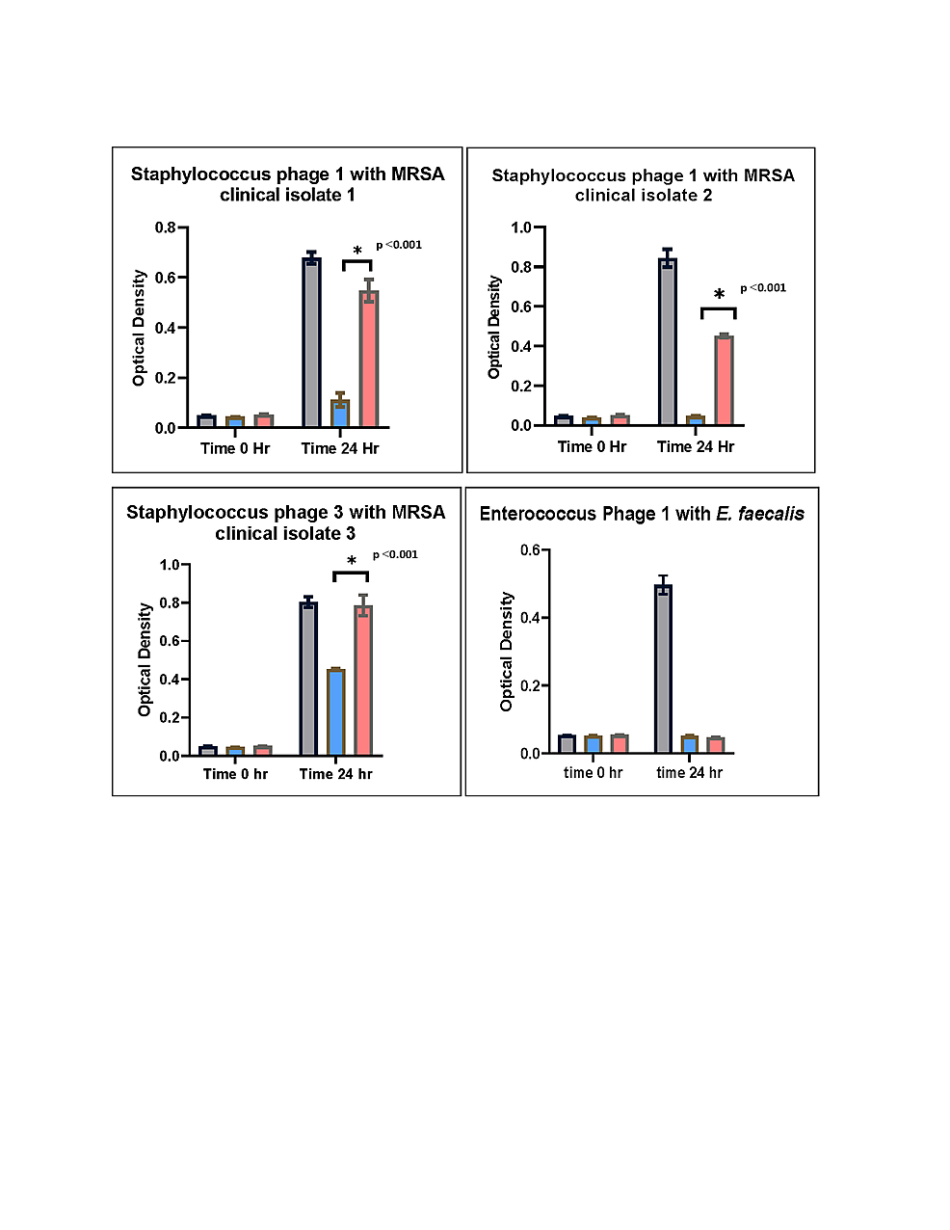
Growth of bacteria and infectivity of bacteriophages in the presence and absence of HP after 24 h of incubation. All had OD of less than 0.1 at 0 h. Gray bars represent bacterial isolates grown in BHI in the presence of HP. Blue bars represent bacterial isolates in BHI without HP but with exposure to corresponding bacteriophages in which drastic bacterial growth inhibition was seen. Red bars represent bacteria in BHI with HP and with exposure to corresponding bacteriophages. Statistically significant reductions in bacteriophage infectivity were seen for all three MRSA bacteriophages compared to infectivity without HP (p value ˂ 0.001). No reduction in the infectivity of Enterococcus phage 1 was observed in the presence of HP. Results are averages of at least eight repeat experiments for each independent bacteriophage-bacteria interaction. Error bars represent standard error. HP, human plasma; OD, optical density; BHI, brain heart infusion; MRSA, methicillin-resistant Staphylococcus aureus

To determine the HP effect on bacteriophage activity, several well-characterized bacteriophages were incubated with their corresponding bacterial isolates including *E. faecalis* and three MRSA strains over 24 h (Figure 1). All four bacteria grew well in BHI broth in the presence and absence of HP (gray bars), but the *E. faecalis* isolate only had OD increase to 0.5. No change in OD was observed when HP, bacteriophages, or normal saline were independently incubated in BHI over 24 h. The growth of all four bacteria strains in BHI without HP was dramatically reduced when bacteriophages were incubated with the corresponding clinical isolates. However, Staphylococcal phage 3 only inhibited the MRSA clinical isolate 3 growth by 42% over 24 h (blue bars). When all three MRSA clinical isolates were independently grown in the presence of HP, there was a significant reduction in Staphylococcal bacteriophage infectivity (p < 0.001). This reduction in infectivity ranged from 48% and 81% for the two MRSA clinical isolates 1 and 2 infected with Staphylococcal phage 1 to 98% for MRSA clinical isolate 3 infected with Staphylococcal phage 3 (red bars). The infectivity of Enterococcus phage 1 in the presence of HP did not change.

## Discussion

To our knowledge, no previous experiments have been conducted assessing bacteriophage activity in the presence of HP. Significant reductions in bacteriophage activity were seen in this experiment when Staphylococcal bacteriophages were in the presence of HP. The exact mechanism of reduced action is unknown, but we hypothesize this is secondary to plasma proteins binding to the bacteria cell surface, shielding the binding sites of bacteriophages, thereby not allowing for attachment and consequently bacterial lysis. With respect to S. aureus, membrane attached coagulase (clumping factor) is known to react with prothrombin causing the formation of staphylothrombin, which then cleaves fibrinogen to fibrin forming a fibrin coat around S. aureus [13-14]. Other bacteria like Enterococcus spp. do not possess a clumping factor and in this experiment, no reduced activity was observed for the Enterococcus bacteriophage in HP supporting our hypothesis. However, it is also known that human serum has high levels of IgG to teichoic acid [15-16] and it has been proven that Staphylococcal bacteriophages predominantly use teichoic acid for attachment [17]. Consequently, another potential mechanism includes IgG antibodies binding to teichoic acid on S. aureus and preventing bacteriophages from binding to their specific attachment sites on teichoic acid, preventing infection and then lysis. 

As seen here, the reduction was not all or none, but instead, there was heterogeneity in the reduction (Figure 1). Therefore, the masking of receptors may depend on receptor quantities, affinities, and accessibility of bacteriophage to these receptors in the presence of plasma proteins. Based on these results, it does not seem plausible that neutralizing antibodies to the bacteriophage or global inhibitors are responsible for the reduced activity. These claims are supported by the heterogeneity of the inhibition, the lack of inhibition with *E. faecalis*, and given the plasma donor had not received bacteriophage therapy. It is improbable that resistance is the cause for these findings because the bacteriophages significantly inhibited growth without HP over the same time and these results were highly reproducible. Nonetheless, future experiments are needed to clarify the mechanism responsible for this inhibition. 

Regardless of the mechanism, these findings have clinical implications. Bacteriophage therapy has been theorized to be a potential adjuvant to reduce morbidity and mortality in *S. aureus* bacteremia [3]. However, microscopic plasma protein aggregates are an important factor leading to Staphylococcal dissemination and abscess formation, causing high mortality and morbidity [9-10]. However, as seen here, the effectiveness of bacteriophage therapy in reducing these parameters in S. aureus bacteremia may be difficult given the masking of S. aureus bacteriophage binding receptors in the presence of HP. Moreover, in PJIs, it has been shown that plasma protein aggregates can form in the synovial fluid and have been hypothesized to cause chronic infections in PJIs [8, 18]. Several case reports support the potential effectiveness of bacteriophage therapy in treating recalcitrant PJIs when this therapeutic is used as an adjuvant with surgical interventions [1, 19]. Without surgery, there is a paucity of data supporting the effectiveness of bacteriophage therapy for Staphylococcal PJIs [1, 4]. Subsequently, when using bacteriophage therapy in Staphylococcal PJIs, surgical removal of the synovial fluid that harbors these plasma protein aggregates may be paramount to have total activity of this adjuvant therapeutic against chronic infections. While this experiment shows a potential inherent limitation of bacteriophage therapy, further studies are needed to identify the mechanism and evaluate if commonly used pharmaceuticals can prevent or counteract this inhibition. 

This experiment is not without limitations. While all Staphylococcal bacteriophages showed a significant reduction in infectivity in the presence of HP, only three bacteriophages were tested in this experiment. It is not feasible to test the activity of all Staphylococcal bacteriophages, but these findings should be evaluated with other bacteriophage therapeutics to support the findings discussed. This was observed with S. aureus clinical isolates, but not with E. faecalis. The exact mechanism of this observation is unknown and its effects in clinical scenarios are yet to be determined. Therefore, generalizing this finding is impossible at this nascent stage and sequential studies are needed to determine the exact mechanism responsible for this inhibition in activity. In addition, it is known that S. aureus can form aggregates in the presence of plasma proteins and while we observed no observable aggregates in any of the microwells, the OD could have been influenced by the formations of microaggregates. While this is a limitation that can be further evaluated in future experiments with plating and colony counting, the reproducibility of these experiments correlated with the minor standard errors and heterogeneous reductions in phage infectivity support our hypothesis that these findings are not by chance alone. The HP used was from a single donor who had not received bacteriophage therapy and it was not concentrated, thereby theoretically replicating an in vivo environment. However, the translational ability of 10% HP to the in vivo environment is only theoretical and, therefore, further studies are needed to clarify if this reduced activity occurs with other concentrations, with pooled HP, and in other body fluids. In addition, the concentration of IgG to S. aureus teichoic acid was not quantified but should be in future studies to better clarify the mechanism for reduced infectivity.

## Conclusions

In conclusion, bacteriophage therapy may hold promise as an adjuvant in certain infections, but many aspects of this novel therapeutic are still unknown, limiting the ability to design protocols and subsequently conduct reproducible clinical trials. Moreover, this study reinforces our nascent knowledge of bacteriophage activity and stresses the need to conduct translational research in this field which can then be applied to clinical use. This brief report has limitations, but the findings should stimulate further translational research to discover the exact mechanisms for the reduced infectivity, enhancing clinical uses of bacteriophage therapeutics. 
